# Peer Review of “Sexual Health Assessment Is Vital to Whole Health Models of Care”

**DOI:** 10.2196/40023

**Published:** 2022-07-28

**Keywords:** sexual health, sexual health assessment, veteran, health equity, health assessment, whole health model, communication, communication barrier, technological barrier, health care, sexuality, sexual orientation, gender identity, sex, gender, model, care, barrier, well-being, comfort, assessment, EHR, electronic health record, quality, equity

*This is a peer-review report submitted for the paper “Sexual Health Assessment Is Vital to Whole Health Models of Care.”*

## Round 1 Review

### General Comments

This paper's [1] idea is new, but unfortunately, there is no structured format for the abstract or paper. The study methods are not specified, and the conclusion is so short and insufficient.

### Specific Comments

#### Major Comments

1. The study needs a structured format.

2. Please specify different parts of the study such as the methods and results.

3. Are technological barriers just related to the lack of structured data fields for this information within its current electronic health record or note titles?
